# Gender Differences in Patients with Takotsubo Cardiomyopathy: Multi-Center Registry from Tokyo CCU Network

**DOI:** 10.1371/journal.pone.0136655

**Published:** 2015-08-28

**Authors:** Tsutomu Murakami, Tsutomu Yoshikawa, Yuichiro Maekawa, Tetsuro Ueda, Toshiaki Isogai, Konomi Sakata, Ken Nagao, Takeshi Yamamoto, Morimasa Takayama

**Affiliations:** The Tokyo CCU Network Scientific Committee, Tokyo, Japan; University of Miami Miller School of Medicine, UNITED STATES

## Abstract

**Background:**

The clinical features of gender differences in takotsubo cardiomyopathy (TC) remain to be determined. The aim of this study was to evaluate the differences in clinical characteristics of male and female patients with TC.

**Methods:**

We obtained the clinical information of 368 patients diagnosed with TC (84 male, 284 female) from the Tokyo CCU Network database collected from 1 January 2010 to 31 December 2012; the Network is comprised of 71 cardiovascular centers in the Tokyo (Japan) metropolitan area. We attempted to characterize clinical differences during hospitalization, comparing male and female patients with TC.

**Results:**

There were no significant differences in apical ballooning type, median echocardiography ejection fraction, serious ventricular arrhythmias (such as ventricular tachycardia or fibrillation), or cardiovascular death between male and female patients. Male patients were younger than female patients (median age at hospitalization for male patients was 72 years vs. 76 years for female patients; *p* = 0.040). Prior physical stress was more common in male than female patients (50.0% vs.31.3%; *p* = 0.002), while emotional stress was more common in female patients (19.0% vs. 31.0%; *p* = 0.039). Severe pump failure (defined as Killip Class > III) (20.2% vs. 10.6%; *p* = 0.020) and cardiopulmonary supportive therapies (28.6% vs. 12.7%, *p* < 0.001) were more common in male than female patients. Multivariate analysis revealed that male gender (odds ratio = 4.32, 95% CI = 1.41–13.6, *p* = 0.011) was an independent predictor of adverse composite cardiac events, including cardiovascular death, severe pump failure, and serious ventricular arrhythmia.

**Conclusions:**

Cardiac complications in our dataset appeared to be more common in male than female patients with TC during their hospitalization. Further investigation is required to clarify the underlying mechanisms responsible for the observed gender differences.

## Introduction

Takotsubo cardiomyopathy (TC) is characterized by reversible left ventricular dysfunction with predominance in women, especially those who are postmenopausal [1–3]. Data from men with TC are scarce and their clinical features remains to be determined because a large clinical database is lacking. The Tokyo CCU Network database is an ongoing multicenter registry that prospectively collects information from emergency admissions to acute cardiac care facilities [4–7]. This database, including 71 large-volume cardiovascular centers in the Tokyo metropolitan area, provides us a unique opportunity to characterize fundamental feature**s** of the disease. Using this database, our study sought to characterize gender-specific differences in hospitalized patients with TC.

## Methods

### Ethics Statement

Written or oral informed consent was obtained in all cases according to the protocol. Both versions of informed consent were approved by the Tokyo CCU Network Scientific Committee, as the study did not identify individual patients. In addition, the Tokyo CCU Network Registry was an epidemiological annual survey supported by the Tokyo Metropolitan Government. Individual clinical information was recorded into the database by the Tokyo CCU Network members of each institution, and the final datasets were collected by the Tokyo CCU Network Scientific Committee under conditions of anonymity, according to the ethical guidelines on epidemiological surveys released from the Japanese Ministry of Health, Labor, and Welfare.

Participating Hospitals of the Tokyo CCU Network Scientific Committee which contributed to collecting the data of TC: Ayase Heart Hospital; Bokutoh Metropolitan General Hospital; Disaster Medical Center; Edogawa Hospital; Fuchu Keijinkai Hospital; Hakujikai Memorial Hospital; Higashiyamato Hospital; IMS Katsushika Heart Center; Itabashi Chuo Medical Center; Japanese Red Cross Medical Center; Jikei University Daisan Hospital; Jikei University Katsushika Medical Center; Juntendo University Nerima Hospital; Kanto Central Hospital; Kanto Medical Center NTT EC; Kasai Shoikai Hospital; Kawakita General Hospital; Keio University Hospital; Kosei General Hospital; Kyorin University Hospital; Meirikai Chuo General Hospital; Mitsui Memorial Hospital; Musashino Red Cross Hospital; National Center for Global Health and Medicine; National Hospital Organization Tokyo Medical Center; Nihon University Itabashi Hospital; Nippon Medical School Hospital; Nippon Medical School; Tama-Nagayama Hospital; Nishiarai Heart Center Hospital; Nishitokyo Central General Hospital; Ogikubo Hospital; Ome Municipal General Hospital; Saiseikai Central Hospital; Sakakibara Heart Institute; Showa General Hospital; Showa University Hospital; Tama Nambu Chiiki Hospital; Tama-Hokubu Medical Center; Teikyo University Hospital; The Cardiovascular Institute; The Jikei University Hospital; The University of Tokyo Hospital; Toho University Omori Medical Center; Tokai University Hachioji-hospital; Tokyo Heart Center; Tokyo Medical and Dental University; Tokyo Medical University Hachioji Medical Center; Tokyo Medical University Hospital; Tokyo Metropolitan Geriatric Hospital; Tokyo Metropolitan Hiroo Hospital; Tokyo Metropolitan Police Hospital; Tokyo Metropolitan Tama Medical Center; Tokyo Shinjuku Medical Center; Tokyo Women's Medical University Hospital; Tokyo Yamate Medical Center; Tokyo-kita Medical Center; Toranomon Hospital; Toshima Hospital

The review of patient medical records was approved by the Committee of Tokyo CCU Network Scientific Council.

### Study Subjects

A total of 57,783 patients were admitted from 1 January 2010 to 31 December 2012 to the 71 large-volume cardiovascular centers which participated in the Tokyo CCU Network. We examined the records of 368 patients diagnosed with TC, where detailed clinical information was obtained through a secondary questionnaire from the 58 cardiovascular centers.

### Definition of TC

We defined TC according to the following criteria proposed by the Mayo Clinic [8]:

1. Transient hypokinesis, akinesis, or dyskinesis of the left ventricular midsegments with or without apical involvement; the regional wall motion abnormalities extend beyond a single epicardial vascular distribution; a stressful trigger is often, but not always present.
2. Absence of obstructive coronary disease or angiographic evidence of acute plaque rupture.
3. New electrocardiographic abnormalities (either ST-segment elevation and/or T-wave inversion) or modest elevation in cardiac troponin.
4. Absence of pheochromocytoma or myocarditis.

### Baseline parameters

The following patient data were collected:

1. Elapsed time from TC onset to hospitalization.
2. Clinical profiles including chief complaint, age and preceding stresses.
3. Initial vital signs such as systolic and diastolic blood pressure in the emergency room or cardiac care unit, arterial oxygen saturation, heart rate, and Killip classification [9].
4. Electrocardiographic findings on admission, including QT interval (QT prolongation was defined as QTc ≥ 0.44 seconds).
5. Laboratory data on admission, including peak creatinine kinase (CK) during hospitalization. The estimated glomerular filtration rate (eGFR; mL/min/1.73 m^2^) was calculated as “194 x Cr^-1.094^ x age^-0.287^” in men and as “194 x Cr^-1.094^x age^-0.287^ x 0.739” in women [10]; chronic kidney disease (CKD) was defined as an eGFR < 60 mL/min/1.73m^2^.
6. Left ventricular ejection fraction (LVEF) was evaluated by modified Simpson method using echocardiography performed on admission. Left ventricular outflow tract obstruction was considered significant if estimated pressure gradient was > 30 mmHg [11].
7. Cardiac catheterization findings, including coronary angiography, left ventriculography, and endomyocardial biopsy, if performed.

### Clinical Outcomes

We examined the incidence of severe pump failure (defined as Killip Class > III) and ventricular arrhythmic events including tachycardia (VT)/fibrillation (VF), advanced atrioventricular block (AVB), and atrial fibrillation or flutter during hospitalization. We also collected data on therapeutic procedures during hospitalization (such as cardiopulmonary supportive therapies and cardiovascular medications). We defined composite cardiac events as cardiovascular death, severe pump failure, and serious ventricular arrhythmias (such as VT or VF).

### Statistical Analysis

Numerical factors with skewed distribution are shown as median (interquartile range). The Wilcoxon rank-sum test was used to determine statistically significant differences in clinical parameters between two different groups. The chi-square test or Fisher’s exact test were used to compare qualitative variables. Stepwise multiple logistic regression analysis was performed to predict in-hospital composite cardiac events. All statistical calculations were performed using JMP version 10 (SAS Institute, Inc., Cary, North Carolina, USA). A *p* value < 0.05 was considered significant.

## Results

**Fig 1:**
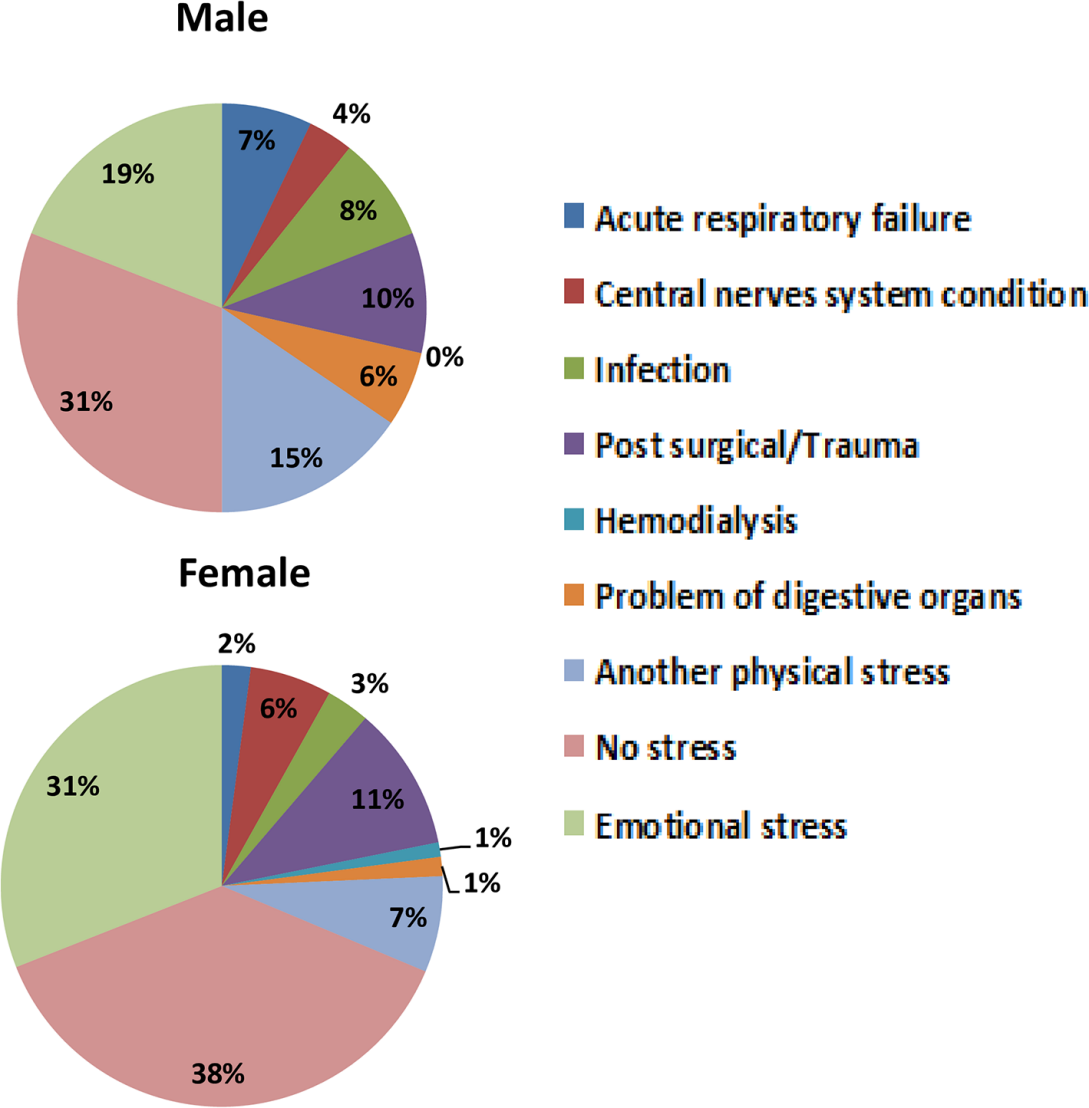
Preceding physical and emotional stresses displayed by gender.

Patient characteristics are shown in Table 1. There were 84 male (22.8%) and 284 female (77.2%) patients. Median patient age was 76 years of age (range: 67–82 years), with male age less than female age. Hospitalization occurred for 86.7% of patients within 24 hours from TC onset, and 92.7% of patients were hospitalized within 72 hours of onset. The chief complaints noted by patients were chest pain (48.6%) and dyspnea (33.4%); chest pain was identified by fewer men (39.3%) than women (51.4%), but the difference was not statistically significant (*p* = 0.051). Preceding physical or emotional stresses were identified in 235 patients (63.9%); physical stress was significantly more common in men than women (50.0% vs. 31.3%; *p* = 0.002), while emotional stress was significantly more common in women than men (31.0% vs. 19.0%; *p* = 0.039). Physical stress is further defined (Fig 1), but we could not obtain a breakdown of the individual content of emotional stress. Vital signs, such as systolic and diastolic blood pressure, heart rate and arterial oxygen saturation were similar between genders.

**Table 1:** Patient Characteristics.

		All patients	Male	Female	*P* value
		(n = 368)	(n = 84)	(n = 284)	
Age (years) [range]		76 [67–82]	72 [64–81]	76 [68–83]	0.040
Hospitalization within 24 hours		86.7%	92.9%	84.9%	0.058
Symptom					
	Chest pain	48.6%	39.3%	51.4%	0.051
	Dyspnea	33.4%	35.7%	32.8%	0.613
Preceding stress					
	No stress	36.1%	31.0%	37.7%	0.260
	Physical stress ^a^	35.6%	50.0%	31.3%	0.002
	Emotional stress	28.3%	19.0%	31.0%	0.039
Vital signs					
	Systolic blood pressure (mm Hg) [range]	133 [111–160]	131 [110–164]	134 [112–160]	0.690
	Diastolic blood pressure (mm Hg) [range]	79 [66–91]	80 [64–92]	79 [67–91]	0.719
	Heart rate (bpm) [range]	87 [75–108]	88 [72–114]	87 [75–104]	0.921
	Arterial oxygen saturation (%) [range]	98 [95–99]	98 [94–99]	98 [95–99]	0.857

^a^ Physical stress included acute respiratory failure, central nervous system disorders, infection, post-surgery, trauma, etc.

Electrocardiographic findings, echocardiographic findings, and laboratory data are shown in Table 2. There were no differences between men and women in terms of electrocardiographic abnormalities. Echocardiographic findings showed that apical ballooning type was noted in 334 patients (90.8%), left ventricular outflow obstruction was found in 30 patients (8.2%), and pericardial effusion was noted in 19 patients (5.2%) with no significant differences in occurrence between genders. Left ventricular thrombus was detected in 2 male patients and no female patients. There was a mild elevation of CK, white blood cell count (WBC), and C-reactive protein (CRP) level in the total patient population. Brain natriuretic peptide (BNP) level on admission was also moderately increased. CK on admission and the peak CK level during hospitalization were significantly higher in men than in women (median value comparison at admission: *p* = 0.013; median value comparison at peak: *p* = 0.012). The median eGFR level was lower in men than in women, but the difference was not statistically significant (*p* = 0.052)**.**

**Table 2:** Electrocardiographic findings, echocardiographic findings, and laboratory data.

		All patients	Male	Female	*P* value
		(n = 368)	(n = 84)	(n = 284)	
Electrocardiogram					
	ST elevation	73.9%	71.4%	74.6%	0.556
	Negative T	70.7%	67.9%	71.5%	0.522
	QT prolongation	41.8%	48.8%	39.8%	0.141
Echocardiography					
	Apical ballooning	90.8%	90.5%	90.8%	0.918
	Left ventricular ejection fraction (%)	50 (40–60)	48 (40–60)	50 (40–64)	0.500
	Left ventricular outflow tract obstruction	8.2%	4.8%	9.2%	0.196
	Pericardial effusion	5.2%	6.0%	4.9%	0.710
	Left ventricular thrombus	0.5%	2.4%	0%	0.009
Laboratory data					
	White blood cell count (cells/μL)	8500 (6500–11100)	9100 (7100–11970)	8100 (6400–11000)	0.091
	Creatinine kinase on admission (IU/L)	178 (101–327)	277 (124–535)	141 (96–283)	0.013
	Peak creatinine kinase (IU/L)	286 (156–499)	471 (198–713)	258 (143–394)	0.012
	C-reactive protein (mg/dL)	0.38 (0.1–2.4)	0.56 (0.1–3.0)	0.32 (0.1–2.1)	0.055
	Brain natiuretic peptide (pg/mL)	205 (76–606)	233 (75–521)	199 (76–627)	0.855
	Estimated glomerular filtration rate (mL/min/1.73m^2^)	66.6 (47.1–84.1)	57.5 (36.5–84.7)	69.0 (51.6–83.9)	0.052

Emergency catheterization was performed in 315 cases (85.6%). Coronary angiography was performed in 83.3% of male and 86.3% of female patients, with no significant difference noted between genders (*p* = 0.501). Provocation of coronary spasm using acetylcholine was attempted in only 13 cases (2 men, 11 women), with a positive outcome for 1 woman and negative outcomes in the remaining 12 cases. Endomyocardial biopsy was performed in 2 patients (details unknown). Coronary arterial stenosis was recognized in 31 patients (11 male, 20 female; *p* = 0.061 male vs. female), with single-vessel disease in 22 patients (8 male and 14 female), 2-vessel disease in 7 patients (3 male and 4 female), and 3-vessel disease in 2 female patients. However, wall motion abnormalities were not accounted for by these lesions in any patients.

**Fig 2:**
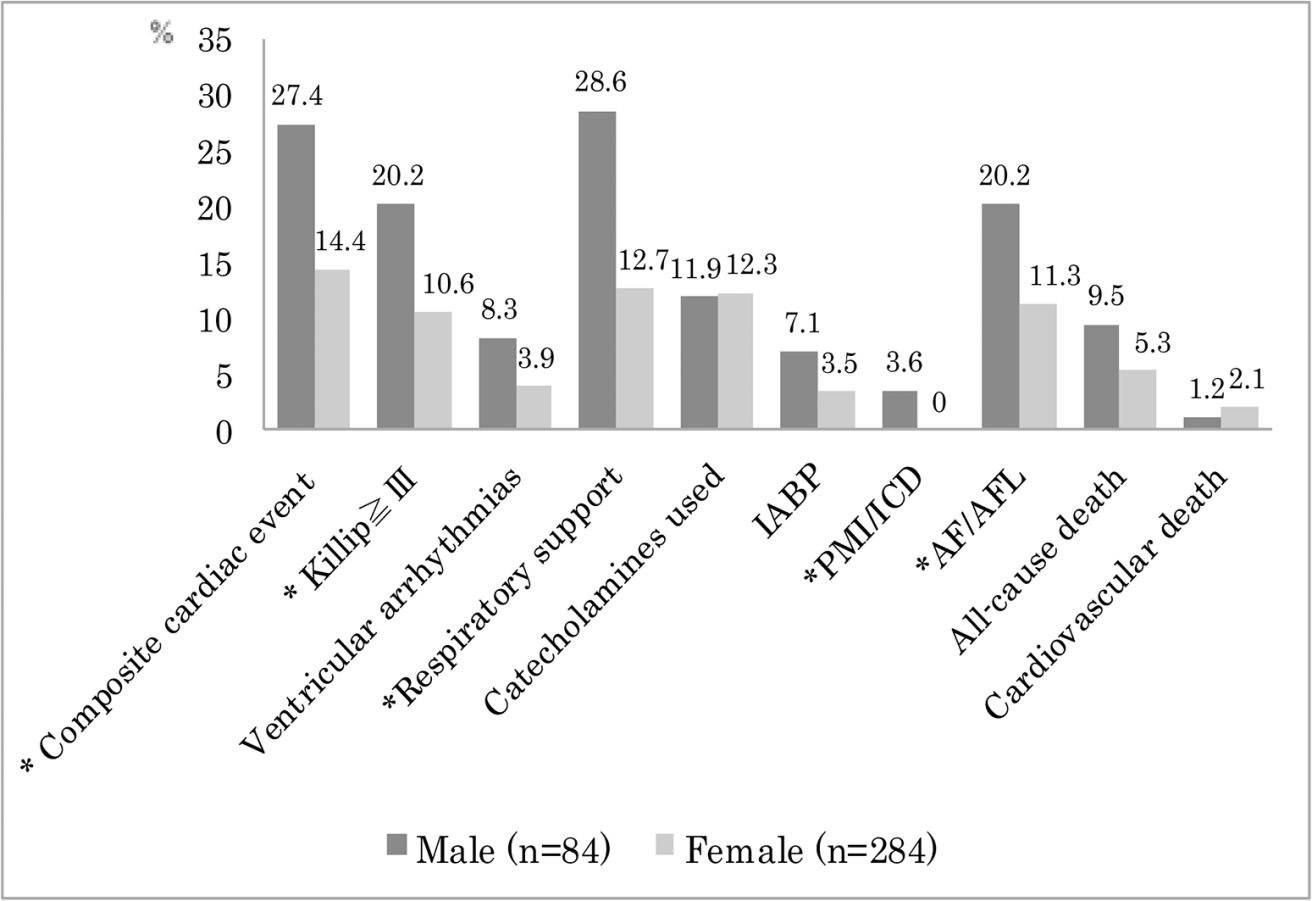
Cardiac complications and cardiopulmonary supportive therapies are shown by gender. Composite cardiac events are defined as cardiovascular death, severe pump failure, or serious ventricular arrhythmias (such as ventricular tachycardia/ventricular fibrillation (VT/VF)). Killip: Killip Class; ventricular arrhythmias: VT/VF; respiratory support: mechanical ventilation or non-invasive positive pressure ventilation; IABP: intra-aortic balloon pump; PMI/ICD: implantation of pacemaker or cardioverter-defibrillator; AF/AFL: atrial fibrillation or flutter. *P value: < 0.05.

Clinical outcome during hospitalization is shown in Fig 2. Three hundred and twenty-one patients (87.2%) were free from severe pump failure, and the remaining 47 patients (12.8%) were complicated by Killip Class ≥ III pump failure. Male patients were more likely to have Killip Class > III pump failure. Serious ventricular arrhythmias tended to be more common in men (non-sustained VT, n = 2; sustained VT, n = 1; VF, n = 4) than women (non-sustained VT, n = 5; sustained VT, n = 4; VF, n = 2), but the difference was not significant. Respiratory support was more commonly needed in male patients with than female. Catecholamines were used in 12.2% of patients and 4.3% of cases received intra-aortic balloon pumps, with no difference between male and female patients. AVB was observed in 4 patients (1 male, 3 female) requiring permanent pacemaker implantation in the male patient. Two male patients received implantation of cardioverter-defibrillators because of VF. Implantation of a permanent pacemaker or cardioverter-defibrillator was more common in men than women (3.6% vs. 0%, *p* = 0.001). Atrial fibrillation or flutter was noted in 17 male and in 32 female patients (20.2% vs. 11.3%; *p* = 0.034).

Twenty-three patients (6.3%) died during hospitalization. There were 7 cardiovascular deaths (1.9%), including 5 heart failure deaths, 1 death due to cardiogenic shock, and 1 death due to cerebrovascular infarction. In the remaining 16 patients, the primary causes of death were non-cardiac, but 50% of patients were complicated by Killip Class ≥ II pump failure.

In-hospital composite cardiac events were noted in 64 patients (Table 3). This cardiac event was significantly more common in male than female patients (35.9% vs. 20.0%, *p* = 0.006), in patients with chronic kidney disease (CKD, *p* = 0.003), and those with prior history of physical stress (*p* = 0.018). WBC count (*p* < 0.001) and BNP values (*p* < 0.001) were higher in patients with cardiac events than those without events. LVEF was lower in patients with cardiac events (*p* = 0.005).

**Table 3:** Differences in clinical variables between patients with a composite cardiac event and those without an event.

Variable	Event Present	Event Absent	*P* value
	(n = 64)	(n = 304)	
Male gender (% of total)	35.9%	20.0%	0.006
Chronic kidney disease present	63.5%	43.8%	0.003
Age (years)	77 (66–83)	76 (68–82)	0.710
White blood cell count (cells/μL)	10900 (8100–15010)	8050 (6200–10500)	<0.001
C-reactive protein (mg/dL)	0.6 (0.11–2.75)	0.3 (0.1–2.2)	0.088
Brain natriuretic peptide (pg/mL)	479 (206–1228)	159 (69–524)	<0.001
Creatinine kinase on admission (IU/L)	180 (120–337)	178 (100–327)	0.644
Left ventricular ejection fraction (%)	44 (35–52)	52 (41–61)	0.005
Physical stress present	48.4%	32.9%	0.018

Stepwise multiple logistic regression analysis was performed using age, gender, CKD, WBC, CRP, BNP, LVEF, and the presence of preceding physical stress. We found that male gender (odds ratio = 4.32, 95% CI = 1.41–13.6, *p* = 0.011), as well as higher WBC (odds ratio = 4.38, 95% CI = 1.38–16.9, *p* = 0.011) were independent predictors of in-hospital composite cardiac events (Table 4). After excluding TC patients with documented coronary artery disease, there were 73 males and 264 female patients; however, in-hospital composite cardiac events still tended to be more common in men than women in this subset of patients with TC (23.3% vs. 14.4%, *p* = 0.069).

**Table 4:** Stepwise multiple logistic regression analysis.

	Odds ratio	95% Confidence	*P* value
Male gender	4.32	1.41–13.6	0.011
Chronic kidney disease present	1.46	0.48–4.84	0.511
High age	1.12	0.36–3.47	0.839
High White blood cell count	4.38	1.38–16.9	0.011
High C-reactive protein level	1.42	0.45–4.70	0.548
High brain natriuretic peptide level	2.61	0.78–9.48	0.119
Low left ventricular ejection fraction	2.09	0.68–7.08	0.198
Physical stress present	0.92	0.28–2.79	0.878

## Discussion

The present study showed that adverse clinical outcomes were more common in male than female patients with TC during hospitalization. We confirmed that male gender was independently associated with composite cardiac events, as was the presence of higher baseline WBC levels.

### Male gender in TC

Previous reports showed that patients with TC were predominantly female, and the percentage of men experiencing TC ranged from 4.4% to 12.7% [12–15]. The Tokyo CCU network database has revealed that 22.8% of the TC patients were male, higher than previous reports. It is of note that the Tokyo CCU Network database consists of patients who were hospitalized at major cardiovascular centers, and does not necessarily reflect the general population with TC. It is well known that emergency ambulance use is greater in men than women in emergency physical situations [16, 17]. Male patients with TC may also utilize such emergency ambulance service sooner and more frequently than females. Actually, percentage of patients who were hospitalized within 24 hours from attack was higher in male than female in the present data. In addition, we analyzed the current data on acute cardiac care in which severely ill patients who were hospitalized to cardiac care unit. In this database, patients with non-cardiac illness who were afflicted by postsurgical stress and infection, etc seem to be excluded. These two factors may be responsible for the discrepancy in the incidence of male gender between Asian and Western countries.

A number of studies have attempted to characterize gender differences in patients with TC [14, 15, 18]. Among them, Brinjikji et al. analyzed a large national inpatient database, and found that mortality was significantly higher in male patients with TC. They concluded that more serious comorbidities might be responsible for the higher mortality in male patients. However, male gender was a still predictor of mortality independently from underlying critical illnesses. In the present study, overall mortality tended to be higher in male than female patients, but was not statistically different. However, composite cardiac events, such as severe pump failure, serious ventricular arrhythmias, and cardiovascular death were significantly more common in male than female patients, suggesting these potentially fatal complications might have contributed to the worse clinical outcomes in male patients. We also found that exaggerated inflammatory response during the process of TC was associated with worse clinical outcome in male patients.

### Coronary artery disease

Previous studies reported that approximately 8% to 10% of TC patients with documented coronary artery disease (CAD) exhibited akinetic areas extending beyond one coronary artery region [1, 2]. In this study, 31 of the 315 patients who underwent cardiac catheterization had CAD. After excluding these TC patients with documented CAD, in-hospital composite cardiac events still tended to be more common in male than female patients. The presence of CAD in these patients did not appear to affect the gender differences noted.

### Role of estrogen

The etiology of TC is not well understood, but a number of previous reports suggested a deficiency of estrogen release as one of the underlying mechanisms for the pathogenesis [19–21], since estrogen exerts various cardioprotective effects including inhibition of excessive sympathoadrenal and renin-angiotensin system activation and antioxidant effects [22, 23]. In ovariectomized rats, myofilament calcium sensitivity was increased in addition to up regulation of beta-adrenergic receptor density [24, 25]. Ueyama et al. reported that increased serum estradiol levels can diminish the pathological changes in the heart induced by emotional stress in an animal model [19]. Reduction of estrogen levels following menopause might be involved as the primary cause of TC both by indirect action on the nervous system and by direct action on the heart [26]. Estrogen treatment also increases the levels of atrial natriuretic peptide and heat shock protein 70 in the heart [27]. Secretion of endogenous estrogen is lower in postmenopausal than premenopausal women, but estrogen level in postmenopausal women appears to be even lower than seen in men [28]. On the other hand, it was reported that during acute stressful events estradiol concentrations become elevated in postmenopausal TC patients compared with an age- and gender-matched control subjects who were afflicted by acute myocardial infarction [29]. During acute onset of TC, higher levels of endogenous estrogen may play a role in mediating cardioprotective effect in female patients with TC, although this “estrogen hypothesis” does not simply account for gender differences in clinical outcomes.

### Role of inflammation

We previously reported that the inflammatory process, as reflected by an increase in WBC, might play a role in the adverse outcomes in patients with TC [6]. Cardiovascular magnetic resonance imaging (MRI) showed complete recovery of left ventricular wall motion abnormality along with inflammatory findings in T2-weighted images [30]. These inflammatory processes may be associated with neurohumoral activation via noradrenaline and BNP [31]. In the present study, there was no significant difference in median WBC values between male and female patients, with a tendency to be slightly higher in men than in women. However, WBC values were confirmed to be an independent predictor of adverse clinical outcome using stepwise multiple logistic regression analysis.

There is limited evidence on how gender affects inflammatory response in cardiovascular disease. Roberts et al. reported that myocarditis induced by Coxsackie virus B3, a prototype of inflammatory cardiovascular disease, affected more male than female mice. They suggested that differential expression of Toll-like receptor signaling might be at least partly responsible for the gender difference seen [32]. Differences in the gene expression profile, coupled with the cardioprotective role of estrogen may be responsible for the gender difference seen in our study.

### Type of Prior Stressors

There was a difference in the prior stressors between male and female patients in the present study, although prior stressors were not identified in approximately one-third of our study patients. Previous reports on small patient populations suggested similar findings [13], and we confirmed this issue using a larger sample size. We cannot exclude the possibility that physical stress affects clinical outcome more than mental stress, however.

### Study Limitations

Not all patients underwent catheterization (85.6% patients were catheterized), so the diagnosis of TC was made according to coronary computed tomography, nuclear medicine scan, or characteristic findings on electrocardiography and echocardiography in the remaining patients who did not undergo catheterization. Biopsy was performed in only two cases and cardiac MRI (which revealed myocardial inflammation) was performed in only 20 cases. Lastly, the Tokyo CCU Network database included limited data during hospitalization for cardiovascular reasons.

## Conclusions

We report gender differences in TC based upon the multicenter Tokyo CCU Network database. Male patients with TC seem to have more serious cardiac complications during hospitalization than female patients. More careful monitoring and more intensive therapies may be required for men with TC than for women with the same condition during hospitalization. Furthermore, it is necessary to further clarify the underlying mechanisms responsible for the noted gender differences.
